# Ischemic heart disease-related mortality trends in the United States (1999–2020) and prediction using machine learning

**DOI:** 10.1097/MS9.0000000000003377

**Published:** 2025-06-10

**Authors:** Noman Khalid, Sabrina Clare Higgins, Muhammad Abdullah, Hasan Munshi, Mahnoor Hasnat, Rajkumar Doshi, Patrick Michael, Rahul Vasudev, Shamoon E. Fayez, Julio A. Panza

**Affiliations:** a Department of Internal Medicine, St. Joseph’s University Medical Center, Paterson, New Jersey, USA; b Department of Internal Medicine, St. George’s University School of Medicine, Grenada, West Indies; c Department of Public Health and Community Medicine, Shaikh Khalifa Bin Zayed Al Nahyan Medical and Dental College, Pakistan; d Department of Cardiology, St. Joseph’s University Medical Center, Paterson, New Jersey, USA; e Department of Cardiology, Westchester Medical Center, New York, USA

**Keywords:** cardiovascular diseases, epidemiology, ischemic heart disease, mortality trends, public health

## Abstract

**Background:**

Ischemic heart disease (IHD) remains the leading cause of mortality globally, contributing significantly to rising healthcare costs. This study aims to analyze trends in IHD-related mortality from 1999 to 2020 using data from the Centers for Disease Control and Prevention’s Wide-Ranging Online Data for Epidemiologic Research (CDC WONDER) database.

**Methods:**

Mortality data from 1999 to 2020 were extracted from the CDC WONDER database, with IHD identified as the primary cause of death (International Classification of Diseases, 10th Revision Codes: I20–I25). Age-adjusted mortality rates (AAMRs) were calculated and analyzed. Trends were assessed using Joinpoint Regression Analysis to determine the annual percentage change (APC). Additionally, predictive modeling was performed using an autoregressive integrated moving average model implemented in Python and Generative Pre-trained Transformer 4.

**Results:**

The study observed a decline in AAMR over the 22-year period, totaling 9 105 056 deaths. The APC revealed a significant decline in mortality rates until 2011 [APC: −4.99, *P* < 0.05, 95% confidence interval (CI): −5.3 to −4.7], followed by a slower decline through 2020 (APC: −2.35, *P* < 0.05, 95% CI: −2.8 to −1.6). Both males (AAMR: 161.4, 95% CI: 161.3–161.6) and females (AAMR: 93.1, 95% CI: 93.0–93.2) experienced a continuous decline in APC until 2018, after which trends began to reverse. African Americans had the highest AAMR (144.1, 95% CI: 143.8–144.3), followed by Whites (125.3, 95% CI: 125.3–125.4), American Indians (106.1, 95% CI: 105.0–107.2), Hispanics (92.9, 95% CI: 92.7–93.2), and Asians (67.1, 95% CI: 66.8–67.4). Geographically, the Mid-Atlantic region exhibited the highest AAMR, followed by the East South-Central region. Among states, Oklahoma had the highest AAMR, followed by New York. Non-metropolitan areas had the highest mortality rates, whereas large-fringe metropolitan areas exhibited the lowest. Predictive analysis suggests a potential plateau or slight increase in mortality rates by 2035 (AAMR: 104.5, 95% CI: 50.05–159.64).

**Conclusions:**

The observed slowing in the decline of IHD mortality rates and the potential for future increases underscore the need for sustained public health interventions and vigilant surveillance to mitigate the burden of IHD.

**HIGHLIGHTS**

- Age-adjusted mortality rates (AAMR) for ischemic heart disease (IHD) decreased from 194 to 91.8 per 100 000, with significant annual declines slowing after 2011.
- Mortality trends varied, with females showing steady declines until 2018, and racial disparities marked by recent increases in annual percentage change among Black, Asian, and Hispanic groups.
- Highest AAMRs were in the Middle Atlantic region and non-metropolitan areas, with projections indicating a slight upward trend in IHD mortality rates by 2035.

## Introduction

Ischemic heart disease (IHD) remains the leading cause of mortality worldwide, accounting for more than 9 million deaths in 2016 alone, making it a major global health concern^[^1^]^. In the United States, IHD is responsible for approximately 30% of all deaths in individuals over the age of 35, highlighting its significant burden on public health^[^2^]^. Although advances in medical therapy, interventional techniques, and public health initiatives have contributed to a decline in IHD-related mortality over the past few decades, the increasing prevalence of cardiovascular risk factors such as obesity, diabetes, and hypertension threatens to offset these gains^[^3^]^.

Beyond its clinical implications, the economic burden of IHD is substantial. IHD-related healthcare costs in the United States alone are projected to rise from $126.2 billion in 2010 to $177.5 billion by 2040, underscoring the urgent need for effective prevention strategies and resource allocation^[^4^]^. Given these alarming trends, there is a critical need for advanced predictive models to assess future mortality rates and guide public health interventions.

Traditional epidemiological models provide valuable insights into disease trends, but they often rely on linear assumptions and may not fully capture the complex interplay of demographic shifts, healthcare interventions, and socioeconomic factors influencing IHD mortality. Machine learning (ML) offers a powerful alternative, leveraging vast datasets to uncover hidden patterns, predict future trends, and inform data-driven policy decisions. By analyzing historical IHD mortality data from 1999 to 2020 using ML techniques, our study aims to provide more accurate forecasts of IHD mortality trends and identify key predictors contributing to disease burden. These insights can aid healthcare policymakers in allocating resources more efficiently and developing targeted interventions to mitigate the impact of IHD in the coming decades.

## Methods

Data for this study were obtained from the Centers for Disease Control and Prevention’s Wide-Ranging Online Data for Epidemiologic Research (CDC WONDER) database, covering the period from 1999 to 2020. We extracted de-identified mortality records related to IHD, focusing on cases where IHD was listed as the primary cause of death. The study population included individuals diagnosed with IHD, classified according to the International Classification of Diseases, 10th Revision (ICD-10), using the following codes: I20 (angina pectoris), I21 (acute myocardial infarction), I22 (subsequent myocardial infarction), I23 (certain current complications following acute myocardial infarction), I24 (other acute IHDs), and I25 (chronic IHD).

Statistical analysis involved the use of Joinpoint Regression to assess temporal trends in IHD mortality and the autoregressive integrated moving average (ARIMA) model to forecast future mortality rates. Joinpoint Regression was implemented using the Joinpoint Regression Program, developed by the National Cancer Institute, to detect significant shifts in mortality trends. This method fits piecewise linear regression models to the data, identifying points where trends change significantly. The optimal number of joinpoints was determined using a Monte Carlo permutation test, ensuring statistical rigor. Trend changes were quantified through annual percentage change (APC) and average APC, with corresponding 95% confidence intervals (CIs) and *P*-values to assess statistical significance.

For predictive time-series analysis, the ARIMA model was selected due to its flexibility in capturing underlying trends, seasonality, and cyclic variations in mortality rates. The model was applied in a structured approach, beginning with an assessment of stationarity using the Augmented Dickey–Fuller test to determine whether differencing was required to stabilize the mean and variance. The autoregressive (*p*) and moving average (*q*) orders were determined based on the Akaike Information Criterion, which minimizes model complexity while maximizing predictive accuracy. The final model was validated through residual diagnostics to ensure adequacy and accuracy in forecasting.

The ARIMA model was implemented using Python’s statsmodels library, a widely used statistical package designed for estimating and analyzing statistical models, hypothesis testing, and data-driven forecasting. statsmodels provides robust tools for time-series analysis, allowing for efficient model fitting, parameter selection, and validation of predictive models. While Generative Pre-trained Transformer 4 (GPT-4) (OpenAI, California) was used solely for assistance in verifying Python code and ensuring statistical procedures were correctly implemented, all modeling and analysis were conducted within statsmodels to ensure methodological rigor, reproducibility, and transparency.

To standardize comparisons, age-adjusted mortality rates (AAMRs) per 100 000 population were calculated using the 2000 U.S. Census population as the reference standard. This adjustment accounted for demographic shifts over time, allowing for meaningful trend analysis across different years.

This study was conducted in accordance with the Strengthening the Reporting of Observational Studies in Epidemiology (STROBE) guidelines, which provide a robust framework for transparent and accurate reporting of observational research. The analysis utilized publicly available, de-identified data from the Centers for Disease Control and Prevention (CDC) Wide-ranging Online Data for Epidemiologic Research (WONDER) database. As the data are fully anonymized and do not contain any identifiable personal information, the study was exempt from institutional review board (IRB) oversight and did not require informed consent, consistent with the U.S. Department of Health and Human Services regulations for the protection of human subjects (45 CFR 46, also known as the Common Rule).

## Results

**Figure 1.**
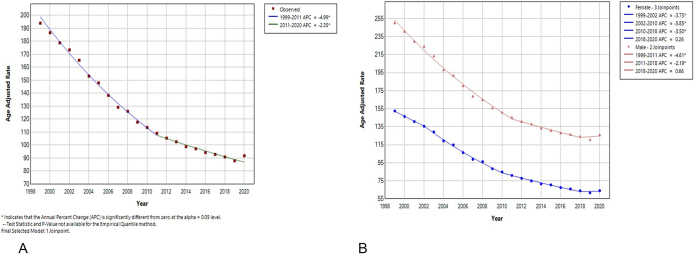
Annual percentage change (APC) of ischemic heart disease (IHD) mortality rates year wise and among different genders in the United States, 1999–2020.

Analysis of IHD-related mortality in the United States from 1999 to 2020 revealed a total of 9 105 056 deaths. A significant downward trend in AAMR was observed from 1999 to 2011, decreasing from 194 to 109.2 per 100 000, with an APC of −4.99% (*P* < 0.05, 95% CI: −5.3 to −4.7) (Fig. 1A). This trend slightly slowed down from 2011 to 2020, with AAMR declining to 91.8 and an APC of −2.35% (*P* < 0.05, 95% CI: −2.8 to −1.6) (Fig. 1A).

Gender-specific trends indicated different patterns. For females, the APC declined steadily until 2018 (Fig. 1B). Among males, a consistent decrease was observed until 2018 (Table 1, Fig. 1B).

**Table 1:** Ischemic heart disease mortality trends among different categories in the United States, 1999–2020

Gender	Year/range (AAMR)	APC	95% CI	
Female	1999 (152.5)–2002 (135.6)	−3.75	−4.8 to −2.1	
	2002 (135.6)–2010 (84.9)	−5.85	−6.9 to −5.5	
	2010 (84.9)–2018 (64)	−3.5	−4.9 to −2.9	
	2018 (64)–2020 (64.1)	0.26	−2.8 to 1.8	
Male	1999 (250.5)–2011 (241.2)	−4.61	−5.2 to −3.7	
	2011 (241.2)–2018 (124.5)	−2.19	−5.4 to −1.6	
	2018 (124.5)–2020 (126.2)	0.66	−2.0 to 2.3	
**Race**	**Gender**	**Years/range**	**APC**	**95% CI**
Asian or Pacific Islander	Female	1999–2014	−5.08*	−6.7 to −2.1
		2014–2018	−3.06*	−6.5 to −1.9
		2018–2020	+2.22	−1.8 to 5.0
Black or African American	Female	1999–2002	−2.56*	−3.9 to 0.05
		2002–2010	−6.25*	−7.5 to −5.8
		2010–2018	−3.75*	−4.5 to −2.9
		2018–2020	+3.62*	0.2–5.3
White	Female	1999–2002	−3.92*	−5.3 to −1.7
		2002–2011	−5.66*	−7.2 to −2.6
		2011–2020	−2.85*	−3.6 to −1.9
Hispanic or Latinos	Female	1999–2005	−4.63*	−5.3 to −2.3
		2005–2011	−6.88*	−9.0 to −6.0
		2011–2018	−3.82*	−5.0 to −2.6
		2018–2020	+3.24	−0.5 to 5.5
American Indian or Alaskan Native	Male	1999–2008	−4.64*	−8.0 to −3.7
		2008–2020	−2.85*	−3.3 to −0.8
Asian or Pacific Islander	Male	1999–2015	−4.49*	−4.9 to −4.0
		2015–2020	+1.06	−0.5 to 3.8
Black or African Americans	Male	1999–2003	−3.09*	−4.15 to −0.09
		2003–2011	−5.238*	−7.2 to −4.7
		2011–2018	−2.438*	−3.6 to −1.4
		2018–2020	+4.258*	0.5–6.3
White	Male	1999–2010	−4.69*	−5.3 to −4.1
		2010–2018	−2.32*	−5.3 to −1.8
		2018–2020	+0.48	−2.1 to 1.9
Hispanic or Latinos	Male	1999–2012	−5.04*	−7.1 to −3.0
		2012–2018	−2.43*	−5.5 to −0.5
		2018–2020	+5.01	−0.7 to 8.4
**Category**	**Subcategory**	**Rank**	**AAMR**	**95% CI**
U.S. Census Division Trends	Middle Atlantic	Highest	143.2	143.0–143.4
	East South Central	2nd Highest	137.3	136.9–137.6
	Mountains	Lowest	100.9	100.6–101.2
U.S. States Trends	Oklahoma	Highest	165.6	164.7–166.4
	New York	2nd	161.8	161.4–162.1
	Tennessee	3rd	156.8	156.2–157.5
Urbanization	Nonmetro	Highest	139.1	138.7–139.4
	Large Fringe Metro	Lowest	115.5	115.3–115.6

AAMR, age-adjusted mortality rate; APC, annual percentage change; CI, confidence interval.

^*^ Result is statistically significant with a *P*-value < 0.05.

**Figure 2.**
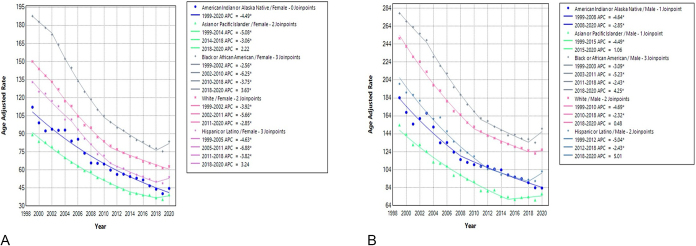
Annual percentage change (APC) of ischemic heart disease (IHD) mortality rates by race in the United States, 1999–2020.

Of note, mortality trends varied by race. Asian or Pacific Islander females and Hispanic or Latino females showed an initial decrease in APC, followed by an increase from 2018 to 2020 (Fig. 2A). Black or African American females consistently decreased in APC till 2018 when an upward trend was seen with APC of 3.63 (*P* < 0.05, 95% CI: 0.2–5.3), while White females demonstrated a steady decline. Among males, American Indian or Alaskan Native and Asian or Pacific Islander groups saw an initial decrease in APC, followed by an increase (Fig. 2B). Black or African American males showed a decrease until 2018, then a significant increase, while White and Hispanic or Latino males displayed a similar pattern with an increase in APC after 2018 (Table 1).

**Figure 3.**
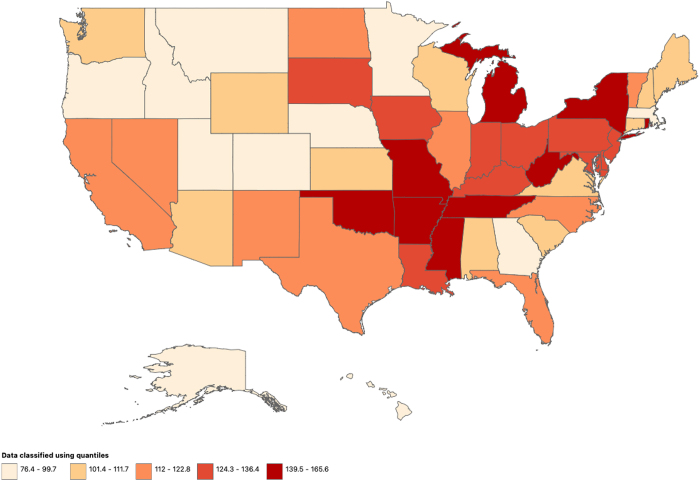
Ischemic heart disease mortality trends (age-adjusted mortality rate) among states of the United States, 1999–2020. Source: CDC WONDER.

Regionally, the Middle Atlantic division reported the highest AAMR at 143.2 (95% CI: 143.0–143.4), followed by East South Central at 137.3 (95% CI: 136.9–137.6), and the lowest in the Mountains division at 100.9 (95% CI: 100.6–101.2) (Supplementary Fig. 1. http://links.lww.com/MS9/A834). State-wise, Oklahoma, New York, and Tennessee had the highest AAMRs (Fig. 3). Non-metropolitan areas had the highest AAMR among urbanization levels, while large fringe metropolitan areas had the lowest (Table 1).

**Figure 4.**
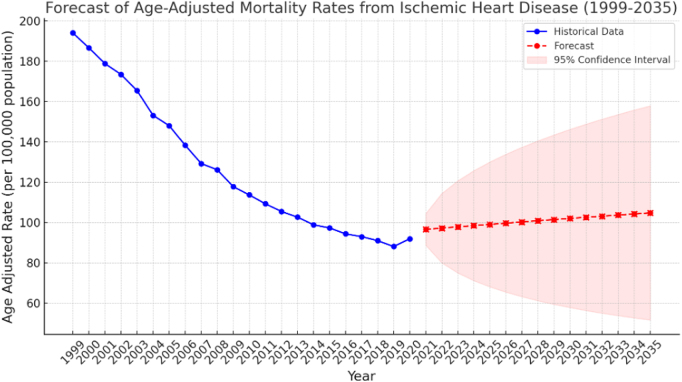
Forecast of ischemic heart disease mortality trends in the United States till 2035.

Figure 4 illustrates the forecast of AAMR from IHD mortality from 1999 to 2035 using an ARIMA model. Based on historical data, there has been a clear downward trend in mortality rates until 2019. The selected ARIMA (1, 0, 1) model demonstrated a satisfactory fit to the data. The Ljung–Box test indicated that the residuals were independently distributed (*P* = 0.08), suggesting that the model adequately captured the information in the time series. The model forecasted a slight increase in the AAMR from IHD in the upcoming years. Specifically, the forecasted rate for 2023 was 97.8 per 100 000 population (95% CI: 74.9–120.6), with a projected increase to 104.8 per 100 000 population (95% CI: 50.0–159.6) by 2035. This projection implies a modest but consistent upward trend in mortality rates (Fig. 4).

## Discussion

Cardiovascular disease is the leading cause of mortality across demographic and gender groups in the United States. One in five deaths in 2021 was due to heart disease, according to the CDC^[^4^]^. This study has consolidated and presented pertinent demographic and geographic variables and their associated mortality trends from 1999 to 2020. Annual, demographic, gender, and geographic region trends all demonstrated a gradual down-trending mortality rate due to IHD over the decade.

Curiously, between 2018 and 2020, the mortality rates for IHD began trending upward across all variables. It could be hypothesized that changes in the social determinants of health within the U.S. population contributed to this rise in cardiac-related mortality. The burden of cardiovascular disease (CVD) has been shown to be influenced directly by social and psychological factors and indirectly by economic stressors^[^5^]^. These may include reduced access to preventive care, increased sedentary lifestyles, and widening income inequality. Healthcare access disparities – particularly in marginalized or rural communities – have long been associated with delayed diagnoses and suboptimal chronic disease management.

The COVID-19 pandemic, a major confounding variable for mortality data from 2020 onward, likely exacerbated many of these social and healthcare disparities. In addition to systemic impacts on healthcare access and delivery, COVID-19 is now well-documented to cause cardiovascular complications, such as myocarditis, heart failure, cardiomyopathy, and myocardial infarction, both during acute illness and in the post-acute phase^[^6^]^. A growing body of literature suggests that the indirect effects of the pandemic, such as interruptions in care for chronic conditions, healthcare worker shortages, and patient hesitancy to seek care, may have contributed to the reversal of long-standing improvements in CVD mortality.

Another consideration for the recent uptrend may be the introduction and widespread use of high-sensitivity troponin, which has led to an increase in the sensitivity of IHD diagnosis and may have influenced mortality attribution^[^7^]^. Additional factors such as an aging population, increasing comorbidity burden, sedentary lifestyles, and increased consumption of calorie-dense processed food continue to shape cardiovascular risk at the population level.

The overall IHD mortality rate showed a significant decline, with an APC of 4.99% (*P* < 0.05) per year until 2011, after which the decline slowed to 2.35% (*P* < 0.05). This change in slope may reflect broader socioeconomic influences. For example, the period following the Great Recession (ending in 2009) has been associated with prolonged recovery and rising healthcare costs, both of which can impact preventive care and management of chronic conditions^[^8^]^.

Gender analysis showed that, overall, females had a lower mortality rate than males. Females demonstrated a significant decline from 2002 to 2010, which then slowed until 2018, after which mortality rates began to rise. Males showed a steady decline from 1999 to 2011, which nearly halved from 2011 to 2018 before reversing into an upward trend. Interestingly, male and female trends ran nearly parallel between 2011 and 2018. This parallelism may suggest temporary gains from more equitable access to healthcare or overall societal improvements in diet and economic opportunity. Future research should assess whether gender-focused preventive strategies during that time may have played a role.

Arguably the most clinically significant findings relate to racial comparisons. Our analysis showed that Black/African Americans had the highest AAMR, followed by Caucasians, while Asian or Pacific Islanders had the lowest. These findings align with prior studies that highlight persistent racial disparities in cardiovascular outcomes, driven in part by systemic factors such as healthcare access, structural racism, and social disadvantage^[^9^]^.

Within gender and race groups, trends were consistent. African American females showed the greatest mortality reduction from 2002 to 2010, while African American males experienced the steepest decline from 2003 to 2011. After 2011, the rate of decline slowed until 2018, after which both groups experienced significant increases in IHD-related mortality. Caucasian males saw a steady decline from 1999 to 2010, but this slowed by 2.37% between 2010 and 2018. Caucasian females maintained a consistent decline from 2002 to 2011 and continued declining – though more slowly – until 2020. Notably, from 2018 to 2020, Caucasian males showed a minimal upward trend in mortality, while females of the same race continued a slower decline.

Hispanic females experienced their most substantial decline between 2005 and 2011. Hispanic males had a continuous decline from 1999 to 2012, which then slowed considerably between 2012 and 2018. From 2018 to 2020, both Hispanic males and females experienced an upward trend in mortality. Of all groups that saw an increase in APC, Hispanic males experienced the largest rise, followed closely by African American males. These findings are supported by previous reports showing that Hispanic and Black populations were disproportionately impacted during the COVID-19 pandemic, due to a confluence of biological, socioeconomic, and healthcare system vulnerabilities^[^10^]^.

This comparison raises important questions regarding the roles of genetic predisposition, social conditions, and healthcare access in shaping these trends. For instance, while Caucasians had the highest number of deaths (7 421 338), they only had the second-highest AAMR. In contrast, Black/African Americans experienced the highest age-adjusted rate (951 745 deaths), suggesting the influence of systemic inequities. A larger Caucasian population may contribute to this difference in total deaths, but the disproportionate burden among African Americans highlights the need to investigate contributing factors such as language barriers, health literacy, cultural stigma, and access to care, irrespective of socioeconomic status.

Geographically, the Mid-Atlantic region had the highest IHD mortality rates, while the Mountain region reported the lowest. Interestingly, many Mountain region states – such as Montana, Wyoming, Nevada, and Arizona – are characterized by access to natural environments and lower population density. It is plausible that improved quality of life, lower stress levels, and better access to whole foods may mitigate cardiovascular risk in these areas^[^9,10^]^. On the other hand, Oklahoma had the highest state-level mortality rate, followed by New York. Notably, the highest IHD mortality rates were found in non-metropolitan areas. This suggests that lack of timely access to emergency care and increased distances between regional healthcare facilities remain important contributors to cardiac mortality, especially in rural America.

While the CDC WONDER database offers a large and nationally representative dataset for analyzing mortality trends, certain limitations should be acknowledged. The database, though comprehensive, relies on death certificate data, which may be subject to misclassification or inaccuracies in cause-of-death reporting. Additionally, it lacks granular clinical information such as comorbidities, medication use, diagnostic criteria, and socioeconomic variables, which could provide a deeper understanding of IHD outcomes. These missing clinical variables may introduce unmeasured confounding and limit the ability to assess individual-level risk factors or treatment patterns.

Moreover, although the selection of data was based on standardized ICD-10 coding, potential inconsistencies in coding practices over time or across regions could introduce systematic biases. These variations may impact the comparability of data across years or geographic locations, which is important to consider when interpreting long-term trends.

From a methodological standpoint, the use of the ARIMA model enables robust forecasting based on historical data; however, it assumes that past patterns will persist into the future. This may not account for emerging public health interventions, novel therapies, or sudden shifts in population health behavior, which could affect the accuracy of predictions.

Despite these limitations, this study’s strength lies in its use of a reliable national dataset and rigorous statistical tools to evaluate IHD mortality trends in the United States over a two-decade period. The application of Joinpoint Regression allows for the detection of statistically significant shifts in mortality trends through the calculation of APC and average APC, while ARIMA modeling provides valuable projections that can inform future healthcare planning and policymaking.

## Conclusion

The current study documents a significant decline in mortality rates associated with IHD from 1999 to 2020, but with a slowing downtrend after 2011 and a potential future plateau or increase. The data highlight significant demographic, gender, and regional variations in mortality trends, pointing to the need for targeted public health strategies and healthcare policies that address the disparities in IHD outcomes. Our findings further emphasize the need for a data-driven approach in healthcare policy and research to reduce IHD’s burden and improve patient outcomes.

### Clinical perspective

This study provides important insights into epidemiological trends and demographic disparities in IHD outcomes over a recent 20-year period. It highlights the necessity for clinicians to enhance knowledge regarding demographic and geographic trends in IHD mortality (and morbidity), particularly in at-risk populations, and to tailor patient care and interventions accordingly. Moreover, the study results emphasize the need for a systems-based approach to healthcare that targets interventions to meet the specific needs of communities with elevated IHD mortality rates.

### Declaration of generative AI and AI-assisted technologies in the writing process

During the preparation of this work, the author used ChatGPT-4 for data analysis and proofreading. After using this tool, the authors reviewed and edited the content as needed and took full responsibility for the content of the publication.

## Data Availability

Data are publicly available on the CDC WONDER Database website.

## Research registration unique identifying number (UIN)

This research study does not involve any human subjects, thus does not require registration.
